# Editorial: Vestibular disorders in children

**DOI:** 10.3389/fneur.2023.1142504

**Published:** 2023-02-14

**Authors:** Jun Yang, Yupeng Liu, Qing Zhang, Lisheng Yu, Toshihisa Murofushi, Klaus Jahn, Maoli Duan

**Affiliations:** ^1^Department of Otorhinolaryngology-Head and Neck Surgery, Xinhua Hospital, Shanghai Jiaotong University School of Medicine, Shanghai, China; ^2^Shanghai Jiaotong University School of Medicine Ear Institute, Shanghai, China; ^3^Shanghai Key Laboratory of Translational Medicine on Ear and Nose Diseases, Shanghai, China; ^4^Department of Otorhinolaryngology-Head and Neck Surgery, People's Hospital of Peking University, Beijing, China; ^5^Department of Otolaryngology, Teikyo University School of Medicine, Mizonokuchi Hospital, Kawasaki, Japan; ^6^German Center for Vertigo and Balance Disorders, Ludwig-Maximilians-University, Munich, Germany; ^7^Department of Neurology, Schön Klinik, Bad Aibling, Germany; ^8^Ear Nose and Throat Patient Area, Trauma and Reparative Medicine Theme, Karolinska University Hospital, Stockholm, Sweden; ^9^Division of Ear, Nose and Throat Diseases, Department of Clinical Science, Intervention and Technology, Karolinska Institutet, Stockholm, Sweden

**Keywords:** pediatric, vestibular disorder, recurrent vertigo, vestibular function, cochlear implantation

## Introduction

Due to the limitation of children's expression ability and the lack of diagnostic experience in pediatricians, vestibular disorders in children are often overlooked, and the prevalence of vestibular disorders in children have been underestimated. An epidemiologic study on the American children revealed that prevalence of dizziness and balance problems was 5.3%, and only 29.9% of them received treatment (1). Vestibular disorders in children have left a lot of confusion for clinicians in many years. Causes of vertigo in children include recurrent vertigo of childhood (RVC), vestibular migraine (VM), Meniere's disease, vestibular neuritis, concussion, inflammation and tumors in the central nervous system, hereditary ataxia, and epileptic vertigo, etc. Peripheral vertigo was more common than central vertigo and other causes of vertigo in children. RVC and VM were the most common diagnosis in peripheral vertigo and central vertigo, respectively. However, persistence of RVC attacks during adolescence could lead to a high prevalence of VM (2, 3). There are three major differences between adult and pediatric patients. Firstly, parents often ignore the manifestations of children's vertigo, and think that they are unwilling to cooperate or mischievous. Secondly, due to the limitation of expression ability and, the complexity of vertigo and accompanying symptoms, it is usually difficult for children to correctly express the characteristics of vertigo. Thirdly, the frequency of occurrence is very different between adults and children for a specific etiology. On the other hand, children with sensorineural hearing loss (SNHL) often suffer from vestibular dysfunction (VD) simultaneously. Around 20–85% of children with SNHL are accompanied by either unilateral or bilateral VD, which is usually an independent factor leading to motor retardation or dysplasia. The incidence of vertigo is increasing, which has become a hot topic in clinical research. Therefore, it is necessary to discuss the diagnosis and treatment of vestibular disorders in children separately. This Research Topic “Vestibular Disorders in Children” consists of nine original articles, one systematic review and one review. We summarized these articles within the following categories: Diagnostic Tools, Common Diseases, and Cochlear implantation and Vestibular Function. Further understanding of the prevalence of various types of vestibular disorders in children, their characteristics, and their management through this Research Topic will benefit pediatric patients and their families, thus decreasing the economic load for the society.

## Diagnostic tools

Vestibular function tests have a great diagnostic value in children with vestibular impairment or vertigo. Vestibular evoked myogenic potential (VEMP) is a myogenic potential recorded on the surface of sternocleidomastoid muscle, eye muscle and other muscles under the condition of strong short sound and vibration stimulation. It is generally believed that the neck muscles of 6-month-old infants have sufficient muscle tension to control head movement, and the results of cervical VEMP (cVEMP) tests at this stage are reliable. Ocular VEMP (oVEMP) can be completed in children over 3 years old (4). Shen et al. concluded that the air conduction and bone conduction cVEMP eliciting rates of 3-month-old infants with normal hearing were 88.89% and 100%, respectively, indicating that stable cVEMP waveforms could be obtained at 3-month-old. They also compared the cVEMP characteristics of 3-month-old sensorineural hearing loss (SNHL) infants and normal hearing infants of the same age. The results showed that the elicited rate of air conduction cVEMP in the SNHL group was lower than that in the normal hearing group. Thereby, they raised the feasibility that cVEMP might be a reliable screening tool for vestibular function in infant. Xiao et al. investigated the effects of acoustic stimulation intensity on oVEMP and cVEMP responses elicited by air-conducted sound (ACS) in healthy children. They concluded that 121 dB SPL can be considered a safe stimulus level for children aged 4–10 years for VEMP testing, while reducing noise exposure. The two papers indicated that VEMP is a non-invasive and well-tolerated test and the parameters established in these studies can provide a reference for the promotion of clinical vestibular function tests.

## Common diseases

The most common diseases causing dizziness and vertigo in children are recurrent vertigo of childhood (RVC) and vestibular migraine (VM). The pathogenesis of RVC is still unclear. According to the diagnostic criteria of Barany society, the diagnosis of RVC is an exclusion criterion of clinical symptoms, lacking the support of other clinical examinations (5). Therefore, high-quality clinical researches on the pathogenesis, clinical features, treatment, and prognosis of RVC are critical for better understanding of such disease entity. Dunker et al. summarized the clinical features and prognosis of RVC in a study of 42 cases. They concluded that age of onset is later and the frequency of attacks is higher in female patients. 45.8% of patients had spontaneous remission of symptoms after 3.5 years of follow-up. The frequency of RVC can be significantly reduced with the correct preventive measures. The study also indicated that few RVC patients showed pathologic findings in ocular motor examinations, head impulse test and VEMP. However, Sun et al. first applied galvanic vestibular stimulation (GVS) VEMP in the research of RVC. They founded that the latencies of ACS-cVEMP and GVS-cVEMP in RVC patients were prolonged compared with normal children. This result suggested that there may be potential damage to the inferior vestibular nerve and its subsequent nerve conduction pathways in RVC patients. They speculated that the retro-labyrinthine portion and lower brainstem along the sacculo-collic reflex pathway were impaired in RVC patients. Rehabilitation is important in RVC patients with vestibular function impairment. Li et al. evaluate the effectiveness of Vestibulo-Ocular Reflex (VOR) adaptation training in RVC patients. They proposed that VOR adaptation training can relieve vertigo symptoms effectively, and it is more acceptable for children when compared with Cawthorne-Cooksey training.

VM is the second most common vestibular disorder in children. Although VM is considered to be a central vestibular disorder, peripheral vestibular organs may also be damaged. Zhang et al. investigated the damage of peripheral vestibular organs in 22 VM children. The results revealed that the superior vestibular nerve and its nerve conduction pathway are possibly damaged in some of the patients. Li et al. concluded that when compared with RVC patients, children with VM younger than 12 years old are more dependent on visual signals when maintaining body balance, and their central nervous system have poorer ability to integrate surrounding information. Episodic ataxias (EA) is a less frequent vestibular disorder in children than RVC and VM. Overlap syndromes among EA, RVC and VM sometimes make it difficult for the clinicians to get an accurate diagnosis. Filippopulos et al. proposed a diagnostic criterion which can help clinicians identify EA patients in children and adolescents. However, the sensitivity and specificity of the criterion need to be further investigated. Concussion may also lead to vestibular syndromes including dizziness and balance impairments. Alkathiry et al. confirmed that The Gait Disorientation Test (GDT) can help clinicians to distinguish between children with concussion and healthy children.

## Cochlear implantation and vestibular function

Due to the close anatomical relationship between the cochlea and vestibule, cochlear implantation (CI) may affect the vestibular function of patients. In the past years, most clinicians paid more attention to the outcome of speech rehabilitation, however, although not many, some studies have noted the vestibular function of patients. The vestibular function of children is inevitably affected after CI (6). Wu et al. conducted a systemic review with 20 clinical studies on the vestibular function changes in children after CI. The results showed a significant increase in abnormal cVEMP, oVEMP and caloric response. A poor Bruininks-Oseretsky Test 2 score was also observed in children after CI, which indicated that static and dynamic balance were also impaired in these children. Deng et al. made a review on the impact and evaluation of vestibular function in children with CI. They summarized the factors which may be associated with postoperative vestibular function change including gender, age, surgical side selection and electrical stimulation, etc. They also proposed valuable strategies from preoperative evaluation to postoperative intervention for children with CI.

## Conclusion

Vestibular disorder in children has its own characteristic, such as difficulty in taking medical history and difficulty in cooperating with some vestibular function tests, etc., and the history of the disease is very important for clinical diagnosis and treatment. Clinicians should be familiar with the common causes of vertigo in children to make the differential diagnosis and reduce unnecessary supplementary examinations. The basic principle of treatment for children with vertigo is to eliminate the cause, relieve vertigo and other accompanying symptoms, and vestibular rehabilitation.

## Author contributions

All authors listed have made a substantial, direct, and intellectual contribution to the work and approved it for publication.
